# The Effect of Antibiotics on the Nervous System: Importance for Anesthesiology and Intensive Care

**DOI:** 10.3390/antibiotics14060622

**Published:** 2025-06-19

**Authors:** Paweł Radkowski, Julia Oszytko, Kamil Sobolewski, Florian Trachte, Dariusz Onichimowski, Marta Majewska

**Affiliations:** 1 Department of Anaesthesiology and Intensive Care, Faculty of Medicine, Collegium Medicum, University of Warmia and Mazury in Olsztyn, 10-719 Olsztyn, Poland; dariusz.onichimowski@uwm.edu.pl; 2 Clinical Department of Anaesthesiology and Intensive Care, Regional Specialist Hospital in Olsztyn, 10-561 Olsztyn, Poland; 3 Hospital zum Heiligen Geist, 34560 Fritzlar, Germany; 4 Faculty of Medicine, Opole University, 45-758 Opole, Poland; oszytkojulia@gmail.com; 5 Department of Microbiology, Faculty of Biological Sciences, University of Wrocław, 50-359 Wrocław, Poland; kamil.roman.sobolewski@gmail.com; 6 Medical Sociology Unit, Hannover Medical School, 30625 Hannover, Germany; trachte.florian@mh-hannover.de; 7 Doctor’s Office, C. Zuleger Extertal, 32699 Extertal, Germany; 8 Department of Human Physiology and Pathophysiology, School of Medicine, Collegium Medicum, University of Warmia and Mazury in Olsztyn, 10-719 Olsztyn, Poland; marta.majewska@uwm.edu.pl

**Keywords:** neurotoxicity, antibiotics, intensive care, GABA inhibition, seizures, complications

## Abstract

Background: Due to the high prevalence of severe infections, antibiotics are frequently administered in anaesthesia and intensive care units. Despite their therapeutic efficacy, several antibiotics exhibit neurotoxic potential, resulting in central and peripheral neurological complications in patients. This review aims to summarise the current evidence on antibiotic-induced neurotoxicity, particularly in critical care settings. Methods: A comprehensive literature analysis was performed to assess the neurotoxic profiles, underlying mechanisms, and clinical manifestations associated with different antibiotic classes, including beta-lactams, fluoroquinolones, macrolides, aminoglycosides, and others. Results: Beta-lactam antibiotics (especially cephalosporins and carbapenems) are strongly associated with seizures, encephalopathy, and EEG abnormalities, mainly through GABAergic inhibition and mitochondrial dysfunction. Fluoroquinolones and macrolides can cause psychosis, insomnia, and neuropathy via NMDA activation and oxidative stress. Linezolid carries the risk of serotonin syndrome and optic neuropathy, while glycopeptides and aminoglycosides are primarily associated with ototoxicity. Risk factors include advanced age, renal or hepatic impairment, and high serum drug levels. Conclusions: The neurotoxic potential of antibiotics is a critical but under-recognised aspect of pharmacotherapy in intensive care. Improved awareness, pharmacovigilance, dose adjustment, and drug monitoring are crucial for mitigating adverse neurological effects.

## 1. Introduction

Antibiotics are among the most commonly used drugs in intensive care units (ICUS). This is because patients there are in a severe condition, which is associated with a weakened immune system. In addition, there is a high probability of contact with pathogenic microbes [1,2,3]. Due to the prevalence of these drugs, adverse reactions have been reported. They occur in 5–10% of hospitalisations, of which 0.1–0.3% are considered severe and may lead to death [4,5,6,7]. The clinical picture and severity of these adverse effects may vary from patient to patient, further complicating their classification. It has been reported that the most observed ARD (adverse drug reactions) are conditions induced by hypersensitivity and allergic reactions, mostly manifested in the form of skin reactions, such as itchiness, nettle rash, and eruption. In more severe cases, vascular oedema or even anaphylactic shock may occur. In some cases, symptoms are limited to individual systems [3]. Neurotoxicity, defined as the predisposition of substances to generate side effects in the central and peripheral nervous systems and sensory organs, is increasingly being identified. However, the underlying mechanism remains poorly understood. The difficulty in correctly interpreting the clinical condition of patients results from the problem of distinguishing ARD from the original disorder. Unusual and non-specific symptoms may be confused with other medications administered simultaneously or with pre-existing diseases [3,7]. In general, drug-induced neurological disorders (DINDS) manifest themselves as headaches, seizures, sleep disturbances, fainting, and cerebral vascular disease [2,8,9,10,11]. Adverse reactions occurring after antibiotic therapy most often manifest in the same way. They tend to go into self-remission, but this depends on rapid intervention and personalised pharmacotherapy [2]. Among the antibacterial drugs that most often stand out in terms of adverse reactions are beta-lactams and quinolones, and cases caused by other antibiotics commonly used in the ICU [3]. Due to the increasing resistance of microbes to the administered pharmacotherapy, specialists are forced to administer larger doses. Initially, toxicity was not associated with high drug concentrations. However, it was observed that in patients with a minimum concentration greater than 64 mg/L for meropenem, 16 mg/L for piperacillin, or 22 mg/L for cefepime could be associated with neurotoxicity in half of them [7,12].

Data on the neurotoxicity of antibiotics used in anaesthesia and the ICU have been overlooked in research. Consequently, the current state of knowledge in this field is largely based on the observations and experiences of other specialists. This article aims to systematise the available knowledge and illuminate the mechanism of pathogenesis of symptoms induced by different groups of drugs directed against bacteria.

## 2. Beta-Lactams

Beta-lactam antibiotics dominate the antimicrobial therapy of patients hospitalised in ICUs, and they are considered to be some of the safest [1,7]. Their mechanism of action consists of inhibiting cell wall synthesis by disrupting the structure of the peptide-glycan, making them broad-spectrum drugs. Depending on their biochemical structure, they can be grouped into five classes: penicillins, cephalosporins, carbapenems, monobactams, and beta-lactamase inhibitors [1,13].

This group of antibiotics, excluding monobactams, is most frequently mentioned in the context of neurotoxicity. It manifests itself in 10–15% of those treated in ICUs in many forms—from confusion and hallucinations to seizures or myoclonia. Renal and brain disorders, advanced age, liver failure, especially metabolic impairments related to cytochrome P450, and all known diseases that increase the permeability of the blood−brain barrier are significant predisposing factors. For example, the penetration of benzylpenicillin is estimated to be 2%, while other antibiotics have the following estimated penetrations: cefazolin at 0.7–10%, cefepime at 10%, and imipenem at 20% [2,14,15,16,17,18]. The time of onset of clinical symptoms varies depending on the beta-lactam used, ranging from 24 h to 30 days [7].

### 2.1. Pathophysiology

The pathophysiological effect of beta-lactam on the nervous system is not precisely defined; however, it can be suspected that various mechanisms are involved, mainly with the action of gamma-aminobutyric acid (GABA) [7], such as reducing its release from the synaptic flask or antagonistic action and inhibition of receptor activity for benzodiazepines (cephalosporin) or non-benzodiazepine (penicillin), due to the structural similarity of the beta-lactam ring to the neurotransmitter. Penicillin maintains the active receptor conformation, which blocks ion conduction. Two types of GABA receptors are described in the literature: GABAA and GABAB. The former is mainly responsible for the convulsive properties of beta-lactams [19]. Cephalosporins (e.g. cefepime and cefazolin) and carbapenems can act specifically as GABA receptor antagonists. Their β-lactam- ring structurally resembles GABA, facilitating competitive or non-competitive binding to the GABA receptor, thereby reducing the GABAergic inhibitory tone in the central nervous system [7,20,21]. In preclinical models, carbapenems such as imipenem and meropenem have been shown to bind at GABA receptor sites via their C2 side chain, and clinical data link elevated cefepime plasma trough levels (>22 mg/L) to seizures and encephalopathy in ICU patients, especially those with renal impairment [7,21].

The epileptogenic potential of beta-lactams is explained by the reduction of inhibitory neurotransmitters and the stimulation of connections entering the cerebral cortex [22,23]. The rationale for this is that in the treatment of seizures induced by beta-lactams, the administration of GABAergic stimulators is more effective than the administration of phenytoin [7]. In addition, the mechanism is distinguished based on the direct influence on human mitochondria, which disrupts their functions, and the release of cytokines and endotoxins attributed to cephalosporin [7,24].

### 2.2. Penicillin

Penicillins, discovered in 1928 by Alexander Fleming, are one of the oldest and most widely used groups of drugs used to combat microbes [25]. Their common component is 6-aminopenicylic acid. It has been shown that alterations in its structure may be responsible for the induction of epileptic seizures. This thesis is supported by the loss of epileptogenic activity after cleavage of the beta-lactam ring [26]. The convulsive potential of penicillin was first documented in 1945. Penicillin G, piperacillin, amoxicillin, ocyline, caricillin, and ampicillin have been implicated in adverse effects in the context of causing nervous system adverse effects (including entanglement, myoclonia, convulsions, and non-convulsive epilepsy (NCSE) [15]. The greatest neurotoxic potential is found with piperacillin and tazobactam, and the first signs of encephalopathy caused by these drugs can be observed between 36 and 168 h after administration [3,15,27,28]. The drug with the most significant convulsive potential is penicillin G, regardless of its concentration in the cerebrospinal fluid (CSF). It binds to the GABAA receptor at the same site as one of the CNS-stimulating compounds [1]. Ampicillin and benzyl penicillin are also attributed to convulsive potentials [3].

Nervous system adverse reactions are most commonly observed after intravenous or intranasal administration of the drug [15]. Vascular administration of penicillin G harms the CNS in adults when the dose exceeds 50 million units within 24 h [3]. A serious consequence of the administration of procaine penicillin to the circulatory system is Hoigne’s syndrome, which occurs in 0.8–16.8/1000 injections [29]. It is thought that the underlying mechanism of this phenomenon involves either congestion of the cerebral vessels or a direct harmful effect of the drug, along with possible stimulation of the limbic system. Symptoms are expressed through hallucinations from all senses, panic attacks, and convulsions, together with concomitant stimulation of the adrenergic system, lasting up to several minutes [3]. Moreover, adverse reactions include abnormalities in the encephalogram (EEG), along with convulsions, confusion, and myoclonia [2,14,16,27,28,29,30]. A short series of β-peaks and polyps and multifocal clonic tugs were observed after amoxicillin administration [31]. Drug-induced aseptic meningitis (DIAM), caused by CD3 lymphocyte stimulation, was observed to increase the likelihood of neurotoxicity by 7.5-fold when the amoxicillin dose exceeded 109.7 mg/L. [2,28,32]. It has been shown that a reduction in the convulsive properties of penicillin G is possible when a sulfonic or amine group is substituted for benzyl hydrogen in its structure [26].

### 2.3. Cephalosporins

Cephalosporins are beta-lactam antibiotics that differ from penicillins in that they combine the β-lactam ring with the thiazolidine ring [33]. They can be divided into five generations due to their spectrum of action [1]. First-generation cephalosporins are characterised by their high activity against Gram-positive bacteria and limited efficacy against Gram-negative organisms. This group includes parenterally administered agents such as cefazolin, cefapyrine, and cephalothin, as well as orally administered agents such as cefradine. Second-generation cephalosporins exhibit greater activity against Gram-negative bacteria while maintaining moderate efficacy against Gram-positive organisms. Examples include cefmetazole, cefotetan, and cefoxitin, which are administered parenterally to treat infections. Cefuroxime is available in both oral and parenteral formulations. Third-generation cephalosporins are primarily active against Gram-negative bacteria and are usually administered parenterally. Agents such as ceftazidime, cefotaxime, and ceftriaxone can cross the blood–brain barrier, particularly the latter two, which makes them useful in the treatment of central nervous system infections and complicated urinary tract infections [13,34,35]. Fourth-generation cephalosporins are used against Gram-negative microbes. These include cefepime, cefiderocol, and cefpirome. Fifth-generation cephalosporins are mainly administered intravenously.

The neurotoxic effects of cephalosporins are similar to those of penicillins, i.e., changes in EEG, altered mental state, or epilepsy [3]. The time to develop neurological symptoms is 1–10 days, and their resolution occurs a few days after discontinuation of treatment [3,36]. They are noticeable after the antibiotic concentration in the bloodstream exceeds 15–20 mg/L. The pathomechanism includes, in addition to inhibition of GABA on many levels, an agonistic action against the N-methyl-D-asparagine receptor (NMDA) [19]. They can occur with drugs of any generation. As of 31 October 2022, no case of neurotoxicity due to the use of fifth-generation agents has been reported in New Zealand [36]. The lowest risk is attributed to cefotaxime and ceftriaxone, and the most common effects are described for cefazolin, cefuroxime, ceftazidime, and cefepime [15,27,28]. The latter is associated with the highest number of reports worldwide and the highest mortality rate [36,37]. This may be because, relative to other cephalosporins, it has a greater affinity for GABA receptors and the possibility of exceeding the blood−brain barrier (BBB) [28]. It is believed to have a higher convulsive potential than that of penicillin [33].

In the context of epileptic complications, cefazolin and cefepime are mainly mentioned, as they have a lower neurotoxicity threshold than other beta-lactams [7,33,38]. The literature reports that neurotoxicity after the use of cefepime occurred in 48% of the cases described when the standard dose was exceeded and in 26% of patients who were selected for the appropriate concentration of the drug [38]. Other specialists observed characteristic changes in EEG in 38% of the subjects, including generalised periodic discharges with triphasic morphology, generalised rhythmic delta activity in 26% of the subjects, and needle and wave patterns in 10% of the subjects. Moreover, the possibility of developing focal seizures has been noted [7]. Cephalexin, a first-generation cephalosporin, exhibited no elliptic activity, which can be attributed to the absence of heterocyclic rings in the 7-aminocephalosporanic acid structure.

Caution should be exercised when administering cefepime to patients with reduced renal clearance, as up to 45% of the serum concentration of this drug exceeds the BBB, in addition to the accumulation of cefepime or its metabolite N-methylpyridine [33].

### 2.4. Carbapenems

Another representative antibiotic containing beta-lactams is carbapenems. Due to their broad spectrum of action and stability against beta-lactamases produced by bacteria, they are considered the most effective [33,39]. Due to the high risk of infection in ICU wards, these antibacterial drugs are most commonly used [1]. Carbapenem-induced neurotoxicity primarily presents as headaches and encephalopathy, with a significantly increased risk of seizures compared to regimens that do not involve these antibiotics [14,15,16,27,29]. Generalised or partial epileptic seizures have been reported following the use of carbapenems, particularly in patients with impaired renal function, where reduced clearance may lead to elevated drug levels and enhanced neurotoxicity [40,41]. However, their severity differs from that of the medicinal products used. The main risks include imipenem, meropenem, doripenem, and ertapenem [3,33]. Their inhibitory effect on the release of valproic acid (VPA) from VPA-Glu in the liver is significant in the pathomechanism of the occurrence of an epileptic attack after carbapenem exposure [1]. Simultaneous administration of antibiotics and VPA results in a 58–88.7% reduction in the concentration of the former and a 50–80% reduction in its half-life [42].

Since the incidence and severity of neurotoxic adverse effects, particularly seizures, differ among specific carbapenems, the following section outlines the current evidence regarding the neurotoxicity of selected agents in this class. Imipenem is the carbapenem with the highest risk of epileptic seizures, estimated at 3–33%. It is usually administered with cefastatin, which inhibits the breakdown of the antibiotic but has not been shown to reduce the seizure threshold [43,44]. The increased frequency of events can be explained by the high affinity for the GABAA receptor and action on the NMDA and AMPA (α-amino-3-hydroxy-5-methyl-isoxazolopropionate complex) receptors [2,33,43]. The use of drugs against Pseudomonas has been observed to increase the risk of seizures [45]. The researchers noted that the use of the drug at a dose of less than 2 g per day, together with weight and kidney problems, did not increase the risk of seizures [43]. Meropenem has a lower epileptogenic potential, with 0.19% of hospitalised patients experiencing epilepsy-like seizures [37]. The low incidence of seizures proves satisfactory tolerability by the CNS. This may be due to the different C-2 side chain structures compared to imipenem [7,33]. Other studies did not indicate any visible differences in the reported side effects [46]. Another antibiotic associated with an increased risk of seizures (0.18%) is ertapenem [40]. Symptoms have been observed in both high-risk groups (renal dysfunction, abnormalities within the CNS) and those without pre-existing conditions [45,47]. Carbapenems with low proconvulsive effects include doripenem and biapenem [48,49]. Experimentally, low risk has been associated with oral tebipenem [44].

### 2.5. Monobactams

Monobactams are antibiotics characterised by a single beta-lactam ring in their structure. The most common is aztreonam, whose neurotoxicity is significantly lower than that of imipenem [1,13,26]. Adverse effects on the CNS are rarely reported, but intraventricular or intraventricular administration has been shown to cause seizures in experimental conditions [33].

### 2.6. Beta-Lactamase Inhibitors

β-lactamase, or enzymes that hydrolyse the amide bond of the beta-lactam ring are produced by Gram-negative bacteria. These are the primary mechanisms of resistance [50]. Neurotoxicity is primarily due to high doses of pharmacotherapy. According to the report, the incidence of events was 10–14%; however, this dose was later found to be overestimated [7]. The reported neurological symptoms include encephalopathy, epileptic states, tonic-clonic seizures, and changes in mental status [51].

## 3. Fluoroquinolones

Fluoroquinolones are representative quinolones, antibiotics used in outpatient treatment and severe hospital infections due to their broad spectrum of action and penetration into body cavities and tissues [3,52]. Their mode of action is characterised by invading the cell through spurts and destabilising the process of unwinding and replicating the nucleic acids of the microorganism by inhibiting the work of enzymes, including topoisomerase IV and II (DNA gyrases) [53,54].

The classification covers four groups of fluoroquinolones: norfloxacin in group I, cyprofloxacin and ofloxacin in group II, levofloxacin in group III, and moxifloxacin in group IV [1]. One of the many known side effects of these antibiotics is peripheral and central neurotoxicity [27]. Symptoms of seizures and headaches caused by norfloxacin, ciprofloxacin, and, less commonly, ofloxacin and levofloxacin occur in 1–2% of intravenous users. The latter is also associated with facial and oral dyskinesia and Tourette’s syndrome. Insomnia, exacerbation of myasthenia (about 24 h after administration), dizziness, and psychosis occurred in 3.7% of intravenous users for at least 48 h, and various extrapyramidal symptoms, including pathological movement, spasmodic movements, and dysarthria [3,53,55]. Despite the widely held view that quinolones lower the seizure threshold, the risk of epileptic seizures is low to very low in adults and children [22,56].

It has been observed that neurotoxicity and psychiatric adverse effects are reported more frequently with third-generation fluoroquinolones compared to second-generation agents, with symptom severity being dose-dependent and typically manifesting within two days of administration [3].

Many factors are behind the appearance of numerous adverse reactions from the nervous system, including the stimulating action of the NMDA receptor, ligand-gated glutamine receptors, and inhibition of GABA [33,57]. Fluoroquinolones exhibit neurotoxic potential via complex interactions with key neurotransmitter systems in the CNS. Additional factors aggravating neurotoxicity include the high affinity of fluoroquinolones for GABAA receptors [58], their structural resemblance to endogenous glutamate receptor ligands, such as kynurenic acid [33], and an increase in oxidative stress that exacerbates neuronal injury. Specific structural groups, such as 7-piperazines (e.g. ciprofloxacin and norfloxacin) and 7-pyrrolidines (e.g. clinafloxacin and tosufloxacin), have been implicated in seizure susceptibility [3,33]. Structurally, these agents act as selective antagonists of GABAA receptors, impairing inhibitory neurotransmission. This effect is attributed to the side-chain substituent at the R7 position of the fluoroquinolone nucleus, which reduces the GABA binding affinity at its receptor site [58,59]. As a result, neuronal excitability increases, which may clinically manifest as seizures, agitation, or altered mental status [58,59]. Animal studies have further demonstrated a significant reduction in brain GABA levels following ciprofloxacin exposure, accompanied by anxiety- and depression-like behaviours in rodents [58]. Simultaneously, fluoroquinolones interfere with glutamatergic transmission via their ability to chelate divalent cations, such as magnesium (Mg^2+^), a physiological blocker of NMDA receptor channels. Chelation of Mg^2+^ prolongs NMDA receptor opening and enhances Ca^2+^ influx, particularly in hippocampal neurones, leading to sustained excitatory neurotransmission [59,60]. This dual effect, the reduction in GABAergic inhibition and potentiation of NMDA-mediated excitation, disrupts the delicate excitatory-inhibitory balance in the CNS, increasing the risk of neurotoxicity. Furthermore, fluoroquinolones can bind to synaptic zinc (Zn^2+^), which normally serves as an endogenous inhibitor of both NMDA and AMPA receptors. Zinc chelation may further potentiate excitatory signalling and contribute to excitotoxicity [61,62]. Prolonged NMDA receptor activation leads to excessive intracellular Ca^2+^ and Zn^2+^ accumulation, triggering oxidative stress, mitochondrial dysfunction and neuronal apoptosis. This excitotoxic cascade may underlie not only the acute neurotoxic effects of fluoroquinolones but also potentially contribute to the progression of chronic neurodegenerative conditions, such as Alzheimer’s disease, amyotrophic lateral sclerosis (ALS), and Parkinson’s disease [58,61,62].

The structure is equally important. 7-piperazines ( ciprofloxacin and norfloxacin) and 7-pyrolidines ( clinafloxacin and tosufloxacin) have been associated with epileptic seizures [3,33]. Antibiotics in this group are effective at penetrating the BBBs, which can cause eosinophilic meningitis [6].

A factor that increases the risk of neurotoxicity is the use of quinolones with other drugs due to their high level of interaction, e.g., the co-administration of NSAIDs or methyloxanthine derivatives increases the convulsive potential [3,33,60].

The literature reports that patients at increased risk of seizures include elderly patients and those with a history of diseases such as kidney or liver failure, seizure episodes, or previous CNS pathologies [3,22,33]. The risk of peripheral neuropathy increases in patients with diabetes [54]. A study by Morales et al. [63] found a 3% increase in the risk of peripheral neuropathy in hospitalised patients with an incident of peripheral neuropathy with each subsequent day of treatment, which persisted up to 6 months after treatment. It has also been observed that the administration of glutamate-binding site agonists of the NMDA receptor (AP-5 or AP-7) has an inhibitory effect on the convulsive effects of these antibiotics [64]. The treatment of side effects caused by fluoroquinolones focuses on restoring the normal function of mitochondria and reducing oxidative stress [43]. Complaints such as neuropathy and general adverse effects on quality of life, such as changes in mental state and disorientation, have been reported with ciprofloxacin use, and there has been a case in which the patient complained of “losing his former self” [53]. Moreover, several studies have reported an increased risk of seizures associated with carbapenem use [46]. Despite its increased ability to penetrate the CNS, ofloxacin is less frequently reported in the context of neurotoxicity [53].

When levofloxacin is used, the most observed complications are seizures and dizziness with psychotic features [57]. There was a case in which this antibiotic caused painful peripheral neuropathy with a pain intensity of 10/10. A skin biopsy confirmed the seizure of small fibres. Fortunately, intravenous immunotherapy reduces the severity of symptoms [65].

## 4. Macrolides

Macrolides are another group of antibiotics with a broad spectrum of action, making them an alternative to penicillins and cephalosporins used in ICU patients [3]. The pathomechanism of their neurotoxicity has not been fully understood, but several mechanisms are possible. It is believed that they include the action of one of the metabolites of clarithromycin—14-hydroxyclarithromycin, altered metabolism of cortisol and prostaglandin, and action on glutamine-receptor and GABA. Additionally, co-administration of other drugs metabolised by the CYP3A4 isoenzyme may lead to elevated plasma concentrations of macrolides, particularly clarithromycin, thereby increasing the risk of neurotoxic effects due to impaired hepatic clearance and accumulation of active metabolites [15,27].

Macrolides have also been shown to activate the integrated stress response, which can lead to cellular dysfunction due to the accumulation of misfolded proteins and subsequent endoplasmic reticulum (ER) stress [66,67]. For example, azithromycin has been reported to inhibit mitochondrial function, resulting in increased reactive oxygen species (ROS) levels, which are known to exacerbate ER stress conditions [66]. The inhibition of autophagic flux by macrolides further complicates their neurotoxic potential, as disrupted autophagy can impair neuronal health and contribute to neurodegenerative processes [67,68].

Azithromycin, erythromycin, and clarithromycin are primarily prescribed to patients with upper respiratory tract infections [69]. After their use, adverse reactions in the form of disorientation, psychosis, insomnia, dizziness, or exacerbation of myasthenia may occur. They most commonly occur within 10 days of drug administration [3]. Another complication is the development of ototoxicity. The occurrence of deafness may resolve, but residues in the form of tinnitus resulting from damage to neurones in the auditory centres of the brain are often observed [70]. Kidney and liver failure, drug overdose, and mental illness are risk factors predisposing to CNS damage [15,27,28].

Additionally, studies have indicated that macrolides can induce inflammation, activating immune responses thereby potentially exacerbating neurological disorders. The activation of pro-inflammatory macrophages as a result of azithromycin treatment has been noted in spinal cord injury models, suggesting a link between macrolide use and adverse outcomes in CNS injuries [68,71]. While these antibiotics exhibit anti-inflammatory properties that can benefit certain conditions, their dual role complicates their use in patients with existing neurological concerns, particularly when dealing with critical conditions in the intensive care unit [71].

Moreover, the application of azithromycin and erythromycin may result in impaired function, dizziness, bilateral hearing impairment, or even loss of hearing. The onset of these symptoms depends on the dose of the medication administered and usually resolves within 14 days of treatment change; however, there have been cases where hearing loss was permanent [3]. No seizures have been observed during azithromycin administration, which is due to the lack of an inhibitory effect on CYP3A4 [22].

Clarithromycin can also induce visual hallucinations. The first reported case occurred after taking it twice daily at 500 mg, 24 h after taking the first dose. A middle-aged patient described their experience as “an ever-evolving landscape of sharks, priests, red lines and other coloured images” [3]. This antibiotic stimulates the transmission of CA3 neurones by reducing the signals from GABA receptors.

## 5. Linezolid

Linezolid is one of the two antibiotics approved for use in the UK from the oxazolidinone group [3]. It exhibits strong activity against Gram-positive bacteria and easily penetrates the CNS due to its lipophilic properties [1,3,72]. It has a strong inhibitory effect on monoamine oxidase (MAO) as it is a reversible, non-selective inhibitor [1,73]. Inhibition of MAOA results in an increase in serotonin (5-HT) levels, while MAOA increases catecholamine levels [3]. Therefore, it should not be combined with drugs that negatively affect serotonin uptake, as this may lead to serotonin syndrome. Other nervous system side effects reported during linezolid use include peripheral and visual neuropathy, dizziness, encephalopathy, and myelosuppression [73].

The most commonly reported neurological symptom is linezolid-induced peripheral neuropathy (LIPN). Its mechanism of action is not fully understood, but it is associated with damage to neuronal mitochondria. It occurs during prolonged antibiotic use above 28 days, with a median of 5 months [3]. LIPN is manifested by changes in body temperature, pain and touch, paraesthesia, or numbness [74]. It is difficult to estimate the frequency of its occurrence, as available studies report values of 6.7–60% [75]. The predisposing factors for the occurrence of these irreversible changes are alcohol abuse, antiviral therapy, diabetes, and previous impairment of the immune system [3].

Furthermore, linezolid-associated optic neuropathy (LION) may be symptom-free or manifest as decreased visual acuity, obscurity, or dyschromatopsia [74]. This is believed to be a result of impaired function of mitochondria located in the optic nerve caused by the use of the drug for more than 28 days [76,77] at doses above 600 mg per day [78]. In most patients, discontinuation of the drug alone results in clinical improvement within a few days, but this process can take six months [73].

Serotonin syndrome (SS) is a potentially life-threatening condition that occurs due to a pathological increase in serotonin levels within the nervous system [74]. Proper diagnosis of this phenomenon remains challenging, as the Sternbach and Hunter criteria—despite their relatively high sensitivity (~84%) and specificity (~97%)—rely primarily on clinical observation and may be limited by the overlap of symptoms with other neuropsychiatric or toxicological conditions, as well as by variability in clinician experience and patient presentation [74,79]. The clinical picture is manifested by a triad of symptoms: psychiatric disorders, neuromuscular activity, and autonomic instability, in addition to hyperthermia [80]. Linezolid, due to its MAO-inhibitory function, may increase serotonin levels, especially when used concurrently with compounds with similar actions [3,74]. In a study involving 230 patients treated with linezolid monotherapy, no SS was reported among 248 adverse reactions [81]. Another study involving 84 studies showed an SS occurrence rate of 0.005% [76]. The concomitant use of linezolid and other drugs that increase 5-HT levels caused SS in 0.11% of patients [74]. Other factors that increase the likelihood of this syndrome include age, low BMI, and kidney or liver failure [82].

## 6. Metronidazole

Metronidazole is a representative nitroimidazole antibiotic that easily penetrates the CNS and has a broad spectrum of action. The reported side effects include pain, dizziness, insomnia, disorientation, and convulsions [33,83]. These symptoms develop slowly and primarily affect hospitalised patients with pre-existing renal or liver failure and pathologies within the CNS [3,84]. Neurotoxicity occurs more frequently when the drug is administered for a prolonged period at a dose greater than 2 g/day [85] or when the total dose exceeds 42 g for treatment lasting more than 28 days [3]. Seizures and other symptoms, in most cases, resolve after discontinuation of therapy [86]. Vascular oedema and cytotoxic, asymmetric changes within the white matter, hyperintensity T2 [33] can be seen on imaging examination. These changes typically resolve within 3–16 weeks after discontinuation of the drug [85]. It is believed that metronidazole-induced neurotoxicity is related to its ability to create free radicals that react with catecholamines, inhibit GABA receptors, or directly affect protein synthesis [3,19]. However, diazepam alleviates the adverse symptoms of CNS [3].

## 7. Glycopeptides

Neurotoxicity associated with glycopeptides may manifest in several forms, including ototoxicity, potential central nervous system (CNS) effects, and hypersensitivity reactions that can trigger systemic inflammatory responses, leading to neurological complications [87,88].

More often, this is due to vancomycin use, and the aetiology of this process is associated with the generation of free radicals, which leads to damage to sensory cells and auditory nerve neurones [89]. Risk factors include old age, use of high concentrations of the drug, kidney failure, and previous hearing problems [3]. Second-generation lipoglycopeptides are not considered to affect the risk of hearing loss or damage.

However, teicoplanin, often regarded as having a favourable safety profile, can induce ototoxicity, characterised by damage to cochlear hair cells, which can lead to hearing loss [90,91]. This effect is particularly concerning in critically ill patients who may require prolonged courses of treatment. Moreover, vancomycin has been linked to CNS side effects, including confusion and dizziness, particularly when high doses are administered or if there is concomitant renal impairment, as it is commonly associated with nephrotoxicity [87,88].

Recent studies suggest that glycopeptides can interact with cellular mechanisms that may lead to neuroinflammation, thereby compounding their neurotoxic potential [24]. Cumulative evidence implicates mechanisms such as oxidative stress and disruption of mitochondrial function, contributing to neuronal injury. Additionally, hypersensitivity reactions commonly associated with glycopeptides, such as Red Man Syndrome, can further complicate their neurotoxicity by precipitating widespread inflammatory responses in the CNS [87,91].

Furthermore, the emergence of newer glycopeptide derivatives with potent antibacterial activity, such as telavancin, raises additional safety concerns; this drug carries a black box warning due to its potential for severe adverse effects, including neurotoxicity, particularly in patients with pre-existing neurological conditions or in elderly patients [92]. Therefore, while glycopeptides are critical components in the treatment of severe bacterial infections, their neurotoxic potential requires careful consideration and robust therapeutic drug monitoring (TDM) strategies to mitigate risks and ensure safe clinical use [93].

## 8. Polymyxin

Polymyximes belong to the antibiotics of natural origin, and their representatives are polymyxin B and colistin [33]. The most common symptoms caused by neurotoxicity in this group are convulsions, diplopia, drooping eyelids, paraesthesia (occurring more often with intravascular than intramuscular administration), convulsions, and muscle weakness [85]. High lipophilicity facilitates drug penetration into the brain, which is associated with a high frequency of side effects [14].

The pathological effect of colistin on the CNS is explained by presynaptic inhibition of the secretion of the neurotransmitter acetylcholine into the synaptic gap. The second possible aetiology is damage to the mitochondria of neurones, which causes the formation of free radicals [84]. The use of colistin may cause neuromuscular blockade (mainly intramuscular), which may lead to apnoea, paraesthesia, or focal seizures (especially with intravenous administration) [33]. Polymyxin B and other antibiotics in this group show increased neuropathological activity [14]. Factors that increase the risk of adverse events include kidney dysfunction, concomitant use of sedatives, drugs, corticosteroids, and hypoxia [27]. Women are more likely to have nervous system damage; however, the reason for this relationship remains unknown [8].

## 9. Aminoglycosides

Aminoglycosides, such as gentamicin, tobramycin, and amikacin, are widely used to treat life-threatening infections caused by aerobic Gram-negative bacteria, particularly in intensive care settings. Despite their clinical efficacy, aminoglycosides are associated with significant neurotoxic potential, primarily in the form of ototoxicity, neuromuscular toxicity, and, to a lesser extent, central nervous system toxicity [77,94].

The hallmark of aminoglycoside-induced neurotoxicity is irreversible ototoxicity, which affects both the cochlear and vestibular systems. Mechanistically, aminoglycosides accumulate in inner ear fluids, where they generate reactive oxygen species (ROS), resulting in oxidative stress, mitochondrial dysfunction, and apoptotic death of inner hair cells [95,96]. Prolonged retention of the drug in the endolymph and perilymph further contributes to sustained cell injury. Clinically, patients may present with high-frequency sensorineural hearing loss, tinnitus, disequilibrium, and vertigo, with damage often progressing even after discontinuation of therapy [95,97,98].

Neuromuscular blockade is another recognised adverse effect, particularly in patients with underlying disorders of neuromuscular transmission or those receiving concurrent neuromuscular blocking agents. Aminoglycosides inhibit the release of acetylcholine at the neuromuscular junction and reduce postsynaptic sensitivity to neurotransmitters, which may result in flaccid paralysis and respiratory depression [99,100]. Risk factors include renal impairment, rapid intravenous administration, and co-treatment with anaesthetics or magnesium sulfate [72,99].

Central nervous system toxicity, although less commonly reported, can occur, particularly with high doses or prolonged therapy. Documented manifestations include seizures, encephalopathy, and altered mental status, especially in patients with underlying CNS pathology or renal dysfunction. The neurotoxic mechanism in the CNS is thought to involve calcium dysregulation, mitochondrial impairment, and possibly N-methyl-D-aspartate (NMDA) receptor-mediated excitotoxicity [101,102].

Genetic predisposition plays a significant role in the development of aminoglycoside-induced ototoxicity. Mutations in mitochondrial 12S rRNA, particularly the A1555G mutation, increase the susceptibility of cochlear cells to aminoglycoside-mediated damage by altering ribosomal RNA and facilitating drug binding, even at therapeutic drug doses. Genetic screening is recommended for specific populations before aminoglycoside use [103,104].

## 10. Conclusions

Antibiotics, commonly used in anaesthesiology and intensive care, can have neurotoxic effects on the central and peripheral nervous systems. The most common adverse reactions include seizures, dizziness, confusion, encephalopathy, and impaired consciousness, the mechanism of which is not fully understood but is associated with the effects on GABA receptors, NMDA, and mitochondrial function.

Beta-lactams, especially penicillins, cephalosporins, and carbapenems, are of particular concern because they can lower the seizure threshold. Fluoroquinolones and macrolides have neurotoxic potential, manifesting as sleep disorders, psychosis, and neuropathy, among others. Linezolid can cause peripheral and visual neuropathy and serotonin syndrome. Other groups of antibiotics, such as glycopeptides, polymyxins, and metronidazole, can also cause serious neurological effects, such as ototoxicity and encephalopathy (Table 1). Risk factors for neurotoxicity include advanced age, kidney and liver failure, high-dose use, and drug interactions. In many cases, symptoms may resolve after treatment is stopped; however, some complications, such as neuropathy, may be permanent. Due to insufficient research on the neurotoxicity of antibiotics, further studies are needed to better understand the pathophysiological mechanisms and develop more effective strategies to minimise the risk of adverse reactions in patients requiring intensive therapy.

Patients on multiple medications face a heightened risk of serious drug–drug interactions, which can compromise treatment outcomes or lead to adverse clinical events, especially in the ICU. Table 2 highlights three well-established high-risk combinations, summarising their mechanisms and clinical implications.

## Figures and Tables

**Table 1:** The most common neurotoxic effects of different antibiotic groups and their mechanisms of neurotoxicity.

Antibiotic Group	Key Representatives	Mechanism of Neurotoxicity	Most Common Neurotoxic Effects
Beta-lactams	Penicillins: penicillin G, piperacillin, amoxicillin, oxacillin, ticarcillin, ampicillin	Inhibition of GABAA receptors (penicillins—non-competitive), reduced GABA release, mitochondrial dysfunction	Seizures, confusion, myoclonus, non-convulsive status epilepticus, encephalopathy
	Cephalosporins: cefazolin, cefuroxime, ceftazidime, cefepime	GABA inhibition (competitive), NMDA receptor activation, mitochondrial toxicity	EEG abnormalities, delirium, seizures, encephalopathy
	Carbapenems: imipenem, meropenem, ertapenem, doripenem	Inhibition of GABA release, blockade of NMDA and AMPA receptors, interaction with VPA	Headache, encephalopathy, seizures (especially imipenem)
	Monobactams: aztreonam	Possible GABA inhibition, poorly understood mechanism	Rare cases of seizures
	Beta-lactamase inhibitors: tazobactam, sulbactam	Possible neurotoxicity due to high doses and metabolic interactions	Encephalopathy, status epilepticus, altered mental status
Fluoroquinolones	Ciprofloxacin, norfloxacin, ofloxacin, levofloxacin	NMDA receptor activation, GABA inhibition, oxidative stress	Seizures, peripheral neuropathy, delirium, psychosis, insomnia,
Macrolides	Azithromycin, erythromycin, clarithromycin	GABA receptor inhibition, altered cortisol and prostaglandin metabolism, drug interactions (CYP3A4)	Disorientation, psychosis, hallucinations, ototoxicity
Aminoglycosides	Gentamycin Amikacin Tobramycin	generation of reactive oxygen species, inhibition of ACTH, and possibly NMDA receptor-mediated excitotoxicity	Ototoxicity, tinnitus, seizures, encephalopathy, and altered mental status
Linezolid (Oxazolidinones)	Linezolid	MAO inhibition, excessive serotonin accumulation, mitochondrial toxicity	Serotonin syndrome, peripheral and optic neuropathy,
Metronidazole	Metronidazole	Free radical formation, GABA receptor inhibition, protein synthesis disruption	Disorientation, headache, dizziness, insomnia, rare seizures
Glycopeptides	Vancomycin, teicoplanin	Free radical-induced damage to cochlear sensory cells and auditory neurons	Ototoxicity, tinnitus, dizziness
Polymyxins	Colistin, polymyxin B	Inhibition of acetylcholine release, mitochondrial toxicity	Seizures, paraesthesia, neuromuscular blockade, muscle weakness

**Table 2:** Summary of the three most common-high-risk- drug–drug interactions that trigger neurotoxicity, with pharmacological mechanisms and associated CNS adverse events.

Drug Combination	Mechanism	Clinical Effects	Ref.
Fluoroquinolone + NSAID	Fluoroquinolones antagonise GABAA→ NSAIDs further enhance convulsant activity.	Seizures, tremors, confusion, hallucinations	[105,106]
Linezolid + SSRI/SNRI	Linezolid is a reversible MAOI → co-administration with serotonergic antidepressants elevates serotonin levels.	Serotonin syndrome: agitation, hyperthermia, rigidity	[107]
Cefepime + benzodiazepine	Cefepime crosses BBB and antagonises GABAA; benzodiazepines are used to treat resultant neurotoxicity.	Encephalopathy, myoclonus, seizures—especially in renal dysfunction	[108]

## Data Availability

No new data were created or analyzed in this study. Data sharing is not applicable to this article.

## Abbreviations

The following abbreviations are used in this manuscript:

ARD	Adverse drug reaction
BBB	Blood−brain barrier
GABA	Gamma-aminobutyric acid
NCSE	non-convulsive epilepsy
CSF	Cerebrospinal fluid
EEG	Encephalogram
MAOA	Monoamine oxidase A
MAO	Monoamine oxidase
UTI	Urinary tract infections
DINDS	Drug-induced neurological disorders
CNS	Central nervous system
ROS	Reactive oxygen species
SS	Serotonin syndrome
LION	Linezolid-associated optic neuropathy
LIPN	Linezolid-induced peripheral neuropathy
5-HT	5-hydroxytryptamine receptors/serotonine receptors
DIAM	Drug-induced aseptic meningitis
AMPA	α-amino-3-hydroxy-5-methyl-isoxazolopropionate complex
NMDA	N-methyl-D-asparagine receptor
VPA	Valproic acid
ICU	Intensive Care Unit
ER	Endoplasmic reticulum
ACTH	Adrenocorticotropic hormone
